# Transcription and translation of human F11R gene are required for an initial step of atherogenesis induced by inflammatory cytokines

**DOI:** 10.1186/1479-5876-9-98

**Published:** 2011-06-26

**Authors:** Bani M Azari, Jonathan D Marmur, Moro O Salifu, Yigal H Ehrlich, Elizabeth Kornecki, Anna Babinska

**Affiliations:** 1 Division of Cardiology, Department of Medicine, State University of New York, Downstate Medical Center, Brooklyn, New York 11203, USA; 2 Division of Nephrology, Department of Medicine, State University of New York, Downstate Medical Center, Brooklyn, New York 11203, USA; 3 Program in Neuroscience, College of Staten Island of the City University of New York, Staten Island, New York 10314, USA; 4 Department of Cell Biology and Anatomy, State University of New York, Downstate Medical Center, Brooklyn, New York 11203, USA

## Abstract

**Background -:**

The F11 Receptor (F11R; aka JAM-A, JAM-1) is a cell adhesion protein present constitutively on the membrane surface of circulating platelets and within tight junctions of endothelial cells (ECs). Previous reports demonstrated that exposure of ECs to pro-inflammatory cytokines causes insertion of F11R molecules into the luminal surface of ECs, ensuing with homologous interactions between F11R molecules of platelets and ECs, and a resultant adhesion of platelets to the inflamed ECs. The main new finding of the present report is that the first step in this chain of events is the *de-novo *transcription and translation of F11R molecules, induced in ECs by exposure to inflammatory cytokines.

**Methods -:**

The experimental approach utilized isolated, washed human platelet suspensions and cultured human venous endothelial cells (HUVEC) and human arterial endothelial cells (HAEC) exposed to the proinflammatory cytokines TNF-alpha and/or IFN-gamma, for examination of the ability of human platelets to adhere to the inflamed ECs thru the F11R. Our strategy was based on testing the effects of the following inhibitors on this activity: general mRNA synthesis inhibitors, inhibitors of the NF-kappaB and JAK/STAT pathways, and small interfering F11R-mRNA (siRNAs) to specifically silence the F11R gene.

**Results -:**

Treatment of inflamed ECs with the inhibitors actinomycin, parthenolide or with AG-480 resulted in complete blockade of F11R- mRNA expression, indicating the involvement of NF-kappaB and JAK/STAT pathways in this induction. Transfection of ECs with F11R siRNAs caused complete inhibition of the cytokine-induced upregulation of F11R mRNA and inhibition of detection of the newly- translated F11R molecules in cytokine-inflamed ECs. The functional consequence of the inhibition of F11R transcription and translation was the significant blockade of the adhesion of human platelets to inflamed ECs.

**Conclusion -:**

These results prove that *de novo *synthesis of F11R in ECs is required for the adhesion of platelets to inflamed ECs. Because platelet adhesion to an inflamed endothelium is crucial for plaque formation in non-denuded blood vessels, we conclude that the *de-novo *translation of F11R is a crucial early step in the initiation of atherogenesis, leading to atherosclerosis, heart attacks and stroke.

## Background

The healthy, non-thrombogenic endothelium of the vasculature does not attract nor bind circulating platelets [1-3]. However, following its exposure to proinflammatory cytokines, the non-thrombogenic endothelium becomes activated and converts into a prothrombotic endothelium [3], resulting in a procoagulant state associated with a predisposition to the adhesion of platelets, atherosclerosis and thrombosis. The adhesion of platelets to the activated endothelium was shown to occur in areas highly prone to atherosclerotic plaque development prior to the detection of lesions, and prior to the infiltration and adhesion of monocytes or leukocytes [2,3]. A critical molecule shown to be involved in the process of platelet adhesion to the activated endothelium is the F11R protein, first described by Kornecki et al in 1990 [4]. F11R is the symbol approved by the Human Gene Nomenclature Committee for the F11 receptor protein (GenBank Accession # 207907; NBC #S56749). In 1995, the amino acid sequences of the N-terminus and internal domains of the platelet F11R molecule were detailed [5]. A protein termed JAM, described in 1998 [6] showed correspondingly-identical amino acid sequences to those of the F11R protein, and hence the alias of JAM-A is also provided here. Direct phosphorylation and dimerization of the F11R protein [5,7] were shown following the activation of human platelets by physiological agonists. The cloning of the human F11R gene revealed that this molecule is a cell adhesion molecule, member of the Ig superfamily [8].

Studies of the adhesion of human platelets to cytokine-inflamed endothelial cells (ECs) [9] determined that homophilic interactions between the F11R molecules expressed constitutively on the platelet surface and the F11R molecules expressed *de-novo *on the luminal surface of ECs when stimulated by cytokines, exert over 50% of the adhesive force between these cells. This observation was evidenced by demonstrating the inhibition of the adhesion of platelets to cytokine-inflamed ECs by a recombinant, soluble form of the F11R protein, and by domain-specific F11R peptides with amino acid sequences stretching in the N-terminal region and the 1st Ig fold of the F11R molecule, respectively [10]. Analysis of the F11R gene identified NF-κB binding sites in the promoter region [11], indicating that cytokines, during processes of inflammation, can cause up-regulation of the F11R gene. Yet, both the biochemical and genetic evidence thus far only suggests the involvement of F11R in the adhesion of circulating platelets to the cytokine-inflamed endothelium. In this report we demonstrate directly, by utilizing small interfering F11R RNAs (siRNAs), that F11R plays a critical role in the adhesion of platelets to the inflamed endothelium, an important early step in atherogenesis.

## Materials and methods

### Human endothelial cells and proinflammatory cytokines

Human aortic endothelial cells (HAEC) and human umbilical vein endothelial cells (HUVEC) (frozen vials of 10^6 ^cells) were purchased from Cascade Biologics, Inc., Portland, OR, and grown in Medium 200 containing 1% or 2% fetal calf serum (FCS) (Cascade Biologics, Inc., Portland, OR). For the experiments detailed below, both HAEC and HUVEC at 2^nd ^passage, were treated with purified human recombinant TNFα (100 units/ml) (R&D Systems, Inc., Minneapolis, MN) and/or IFNγ (200 units/ml) (Roche Diagnostics, Mannheim, Germany), maintained at 37°C for the indicated periods of time. In a series of dose-response experiments in which the concentrations of TNF-α and IFN-γ were varied, a concentration of 50 pM TNFα is equivalent to100 units/ml TNF-α, and a concentration of 5.8 nM IFNγ is equivalent to 200 units/ml IFNγ.

### Quantification of F11R mRNA in HAEC and HUVEC by real-time PCR

HAEC and HUVEC endothelial cells were grown to confluence and treated with cytokines at various times and doses. The treated cells were washed with 1× PBS, lysed, the total RNA extracted utilizing RNeasy Mini Kit (Qiagen, Valencia, CA, USA), and analyzed by real-time PCR on three separate experiments conducted in triplicate. The levels of F11R mRNA were determined by use of an ABI Prism 7000HT Sequence Detection System (ABI; AppliedBiosystem, Foster City, CA). The F11R primers consisted of the forward primer - 740: CCG TCC TTG TAA CCC TGA TT, reverse primer - 818: CTC CTT CAC TTC GGG CAC TA and probe -788: TGG CCT CGG CTA TAG GCA AAC C. The GAPDH forward primer - 620: GGA CTC ATG ACC ACA GTC CA, reverse primer - 738: CCA GTA GAG GCA GGG ATG AT, and the probe - 675: ACG CCA CAG TTT CCC GGA GG. Thermal cycles consisted of: 1 cycle at 48°C for 30 min, 10 min at 95°C and 40 cycles for 15 sec at 95°C, 1 min at 60°C. The probes were dual-labeled with FAM-TAMRA, obtained from ABI. Each mRNA level was expressed as a ratio to GAPDH. The mRNA levels were calculated using a standard curve of RNA isolated from normal human kidney (Stratagene) for the time course and dose curve or QPCR Human Reference total RNA (Stratagene) utilizing the ABI Prism 7000 SDS Software (Applied Biosystems).

### Statistical analysis for real-time PCR

The RNAs, derived from ECs grown and treated in tissue culture wells, were isolated individually. Real time PCR procedures were performed in triplicate and averaged for each sample in three separate experiments (n = 9). The data were analyzed by Student's t-test and by mixed linear model analysis using SPSS software. Differences were considered significant at P < 0.05.

### Preparation of inhibitors of RNA synthesis, NF-κB and JAK protein kinase

Actinomycin D (Sigma, St. Louis, MO), a known inhibitor of RNA synthesis, was diluted in DMSO to a 500 μg/ml (100X) stock solution. Parthenolide (Sigma), an inhibitor of the nuclear factor kappa B, NF-kB signaling [12], was diluted in chloroform to a 50 mM (1000X) stock solution. The inhibitor of Janus kinase, JAK protein kinase, the tyrosine kinase inhibitor tyrphostin AG490 [13], (Sigma) was diluted in ethanol to a 5 mM (100X) stock solution. All stock solutions were diluted in culture media to 1X concentration prior to experimentation. HAEC and HUVEC were grown to confluence and then treated with either actomycin D, parthenolid, or AG490, added in culture media without growth factor supplements for 1 hr at 37°C. Proinflammatory cytokines, TNFα and/or IFNγ were then applied to the media and the ECs were further incubated at 37°C for up to 24 hrs.

### Silencing of the F11R gene of HAEC and HUVEC endothelial cells: transfections with small interfering RNAs (siRNAs)

Transfections were *performed *using Oligofectamine (Invitrogen, Carlsbad, CA) according to the manufacturer's instructions. Briefly, 9 × 10^4 ^HAEC and HUVEC cells were seeded onto 96 well plates in 200 M media supplemented with LSGS without antibiotics, and the transfections of ECs were carried-out with either the stealth F11R siRNA HSS121425 (5'GGGACUUCGGAGUAAGAAGGUGAUUU 3') (300 nM) or the control, non-targeting siRNA No. 2 (Dharmacon). Subsequently, the transfected ECs were incubated in 200 M media containing 1% FBS followed by the application of cytokines TNFα (100 units/ml) and/or IFNγ (200 units/ml) for various periods of time.

### Analysis of F11R in HAEC and HUVEC lysates and cell culture media

Monolayers of arterial and venous endothelial cells (90 - 95% confluence) were collected and homogenized in lysis buffer containing 20 mM Tris, 50 mM NaCl, 2 mM EDTA, 2 mM EGTA, 1% sodium deoxycholate, 1% Triton X-100, and 0.1% SDS, pH 7.4 supplemented with protease and phosphatase inhibitors (Sigma-Aldrich) for the preparation of total cell lysate material derived from human arterial and venous endothelial cells. Protein concentration was quantified by the bicinchoninic acid (BCA) assay. Procedures utilizing SDS-polyacrylamide gel electrophoresis (10%, PAGE) followed by immunoblotting were performed as described previously [14].

### Collection and analysis of F11R in the media from cultured endothelial cells

The media derived from the arterial and venous, cytokine-treated and nontreated endothelial cells were collected at the time of cell harvesting and concentrated 200X using the centrifugal filter Centricon YM-10. Identification of the F11R protein within the collected media involved the resolution of all proteins by SDS-PAGE (10%) followed by immunoblotting procedures utilizing anti-F11R antibody, as described previously [10].

### Quantitation of immunoblots

Quantitation of the immunoblots was performed using image J (NIH). Briefly, scanned images of immunoblots were opened in image J, the protein bands were selected using the freeform tool and measured for integrated density. The values were normalized to tubulin levels by dividing the integrated density of the specific band by the integrative density of the tubulin band. ANOVA statistical analysis was performed on the normalized values. All values are the average of three immunoblots ± SEM.

### The adhesion of platelets to endothelial cells: labeling of human platelets by calcein

Platelet rich plasma (PRP) was prepared from 100 mL of citrated whole blood, by centrifugation at 200 × g for 20 min at 23°C. Calcein (2 μg/mL)(Invitrogen) [15,16] was added to the PRP, and the PRP was maintained at 30°C for 1 hr in the absence of light. Platelets were isolated from PRP, washed as detailed previously [10] and resuspended at final concentrations ranging from 2.5 - 3.5 × 10^8^/mL

Assays conducted for measuring the adhesion of platelets to endothelial cells were performed in the dark due to the sensitivity of the calcium probe calcein to light exposure. Initially, HAEC and HUVEC, plated in cell culture wells, were incubated with 1% FBS/BSA in 200 M media for 1 hr at 37°C to block nonspecific binding sites. Aliquots of freshly-prepared, calcein-labeled platelets (3.3 × 10^8^/ml) were added to each of the cell-culture wells, and plates were incubated at 37°C for 1 hr. Paraformaldhyde (4%), pH 7.4, was added to each well and incubation continued at 23°C for 15 min. The addition of paraformaldehyde, before washings, did not affect the natural capacity of the platelets to adhere to endothelial cells. The plates were washed 3× with pre-warmed growth factor-free 200 M media. Then aliquots (100 μl) of pre-warmed PBS were added to wells, and wells were read using a Perkin Elmer plate reader Victor 3, 1420 multilabel counter with fluorescein filter, as detailed previously described [9].

### Statistical analysis performed for assays involving platelet adhesion to endothelial cells

To improve normality of distribution, the dependent variable (number of platelets per endothelial cell) was transformed by dividing by 10, adding 1 and taking the natural log. A mixed linear model was constructed that introduced treatment, cell type and the state of platelet activation (nonactivated vs agonist-activated) (and their mutual interactions) as fixed factors, with plate as a random factor. Since the variance of the dependent variable differed substantially according to plate, treatment and platelet state, variances were estimated separately for each combination of these factors. Due to the unbalanced nature of the study design, Satterthwaite adjustments were applied to numerator degrees of freedom. To offset the issue of multiple testing, Tukey-adjustments were applied to *p*-values for pair-wise group comparisons. Analysis of model residuals was undertaken to check for model fit and outliers. SAS Release 9.3 (SAS Institute, Cary NC) PROC MIXED software was used. Four outlying observations were excluded from analysis. All of the fixed main effects and their interactions were statistically significant at the 0.001 level, with the exception of the cell type main effect (*p *= 0.783). Discrepancies of means among the 11 plates were significant (Z = 2.11, *p *= 0.017). The inter-assay coefficient of variance was 0.7 ± 0.3 (S.E). The intra-assay coefficient of variance for each condition on the same plate was lower [(range from 0.05 to 0.16 ± .02 (S.E.) (Z > 6.00, P < 0.0001)] than the inter-assay coefficient of variance.

## Results

### Expression of F11R mRNA in human aortic (HAEC) and umbilical vein (HUVEC) endothelial cells exposed to pro-inflammatory cytokines: time and dose-response

**Figure 1:**
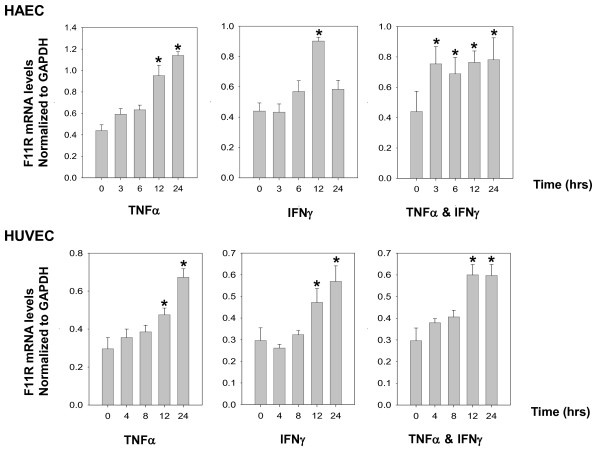
**Expression of F11R mRNA in human aortic endothelial cells (HAEC) and umbilical vein endothelial cells (HUVEC) exposed to proinflammatory cytokines TNFα and/or IFNγ: time course**. Real-time PCR was performed in cultured HAEC (top panels) treated for 0, 3, 6, 12, and 24 hrs with TNFα (100 u/mL) and/or IFNγ (200 u/mL), and in cultured HUVEC (bottom panels) treated for 0,4,8,12, and 24 hrs with TNFα (100 u/mL) and/or IFNγ (200 u/mL). Real-time PCR was performed three times in triplicate for each time point. Values represent the mean ± SEM. *P < 0.05 indicates the level of significance determined at a specific interval of time of cytokine- treatment of ECs in comparison to the zero time points.

The expression of F11R mRNA was examined both in arterial HAEC and venous HUVEC following their exposure to the pro-inflammatory cytokines TNFα and IFNγ. As shown in Figure 1, a time-dependent increase in F11R mRNA expression was observed following the exposure of arterial and venous cells to TNFα or IFNγ, or their combination. Arterial endothelial cells (top panels) demonstrated a slow, significant increase in the level of F11R mRNA at 12 hrs of exposure to either TNFα or IFNγ. Although a further increase was observed with TNFα for a subsequent 12 hr period, further exposure of cells to INFγ resulted in a drop in the F11R mRNA level. The simultaneous treatment of cells with TNFα and IFNγ resulted in a shortening in response time, with maximal F11R mRNA levels observed already at 3 hrs of cytokine-exposure. Similarly, venous endothelial cells (lower panels) demonstrated a gradual enhancement (also significant at 12 hrs) of F11R mRNA expression following the application of cytokines, alone or in combination.

Comparison of the F11R mRNA level in untreated vs cytokine-stimulated endothelial cells indicated that F11R mRNA levels were higher in arterial than in venous ECs, with the overall pattern in the response-time to cytokines similar in both cell types.

**Figure 2:**
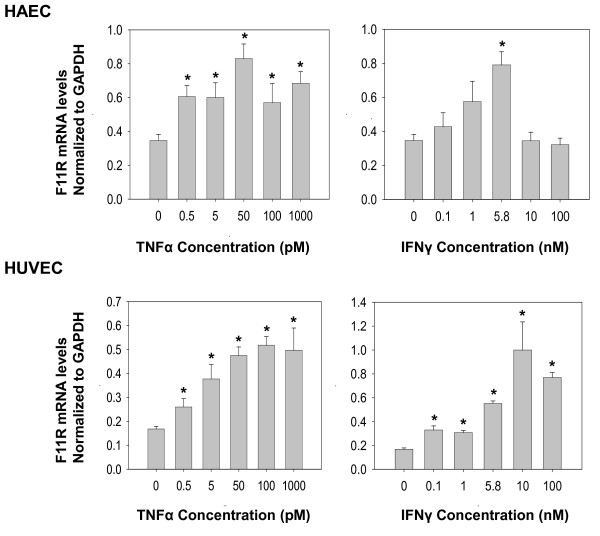
**The expression of F11R mRNA in human endothelial cells (ECs) exposed to proinflammatory cytokines TNFα and IFNγ: dose response**. Endothelial cells, HAEC and HUVEC *in culture*, were treated with different concentrations of TNFα (0.5 to 1000 pM; 1 to 2000 units) and IFNγ (0.1 - 100 nM; 3.4 - 3448 units/ml) for 12 hrs at 37°C. Real-time PCR was performed three times in triplicate for each time point. Values represent the mean ± SEM. * P < 0.05. Significant differences in F11R mRNA observed at the indicated concentrations of cytokines in comparison to levels of F11R mRNA measured in the absence of cytokines.

By varying the concentration of cytokines, the level of F11R mRNA was observed to increase in both cell types, in a dose-dependent manner following a 12 hr exposure to either TNFα or IFNγ. As shown in Figure 2, significant increases in F11R mRNA levels in arterial EC in response to TNFα, already were observed at concentrations of TNFα as low as 0.5 pM (1 unit/ml), with maximal responses to TNFα observed at 50 pM (100 units/ml). In HUVECs, significant increases in F11R mRNA levels in response to TNFα also were observed at a concentration of TNFα of 0.5 pM, whereas maximal increases occurred at a concentration of 100 pM TNF-α (200 units/ml).

Arterial EC exhibited sensitivity to IFNγ already at a concentration of 0.1 nM (3.4 units/ml), with maximal, significant increases in F11R mRNA levels in response to IFNγ at 5.8 nM (200 units/ml). However, the treatment of arterial endothelial cells with higher concentrations of TNFα (of 100 or 1000 pM; 200 or 2,000 units/ml) or IFNγ (10 or 100 nM; 344 or 3448 units/ml), resulted in a drop in the expression of F11R mRNA to pretreatment levels, as was observed with IFNγ (Figure 2, top panels). Similarly, venous endothelial cells demonstrated significant increases in F11R mRNA level in response to TNFα at 0.5 pM (1 unit/ml) and 0.1 nM IFNγ (17 units/ml) with maximal increases occurring at concentrations of 50 pM TNFα (100 units/ml) and 10 nM IFNγ (344.8 units/ml). A ten-fold higher concentration of IFNγ produced a slight decrease in the expression of F11R mRNA in venous endothelial cells, but not a complete drop, as observed in arterial endothelial cells at higher concentrations.

A comparison of the concentrations of cytokines used in this study and the physiological and pathophysiological concentrations of cytokines measured in individuals indicates that serum concentrations of TNFα, found in normal individuals were about 0.8 pM, whereas pathophysiological concentrations of TNFα, 4-fold higher (3.2 pM), were detected in the serum of patients (see the link- http://www.ncbi.nlm.nih.gov/pmc/articles/PMC1533889/table/T1/). As shown in Figure 2, the concentrations of TNFα that significantly induced F11R mRNA in both HAEC and HUVEC were in the same range. Likewise, a concentration of IFNγ, of about 0.1 nM, was reported in the serum of patients (see link above) - a concentration of IFNγ shown to significantly induce F11R mRNA in both HAEC and HUVEC (see Figure 2).

### Inhibition of the expression of F11R-mRNA in inflamed endothelial cells

**Figure 3:**
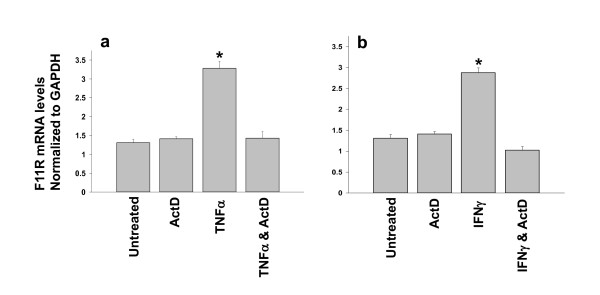
***De novo *expression of F11R mRNA in inflamed endothelial cells: blockade of F11R mRNA expression in endothelial cells treated with TNFα and IFNγ by the RNA synthesis inhibitor, actinomycin**. Confluent monolayers of HUVEC were maintained under Untreated conditions, or pretreated with the RNA synthesis inhibitor, actinomycin D (ActD) (5 μg/mL), in growth supplement-free media for 1 hr at 37°C. The response of HUVEC maintained in the presence of ActD alone is shown in histogram labeled ActD. The response of HUVEC treated with TNFα alone(100 u/ml) is shown in Figure 3a, and the response of HUVEC treated with IFNγ alone(200 u/ml) for 24 hrs is shown in Figure 3b. The response of HUVEC pretreated with ActD prior to 24 hr exposure to either TNFα (100 u/mL) or IFNγ (200 u/mL), is shown in the histograms labeled TNFα & ActD (see Figure 3a) or IFNγ & ActD (see Figure 3b). The F11R mRNA levels were measured by Real-Time PCR in triplicate for each condition. Values are the mean ± SEM. * P < 0.05 significant differences in F11R mRNA observed between cells exposed to TNFα or IFNγ alone vs ECs treated (or not treated) with ActD alone or ECs treated with ActD followed by their exposure to either TNFα or IFNγ.

**Figure 4:**
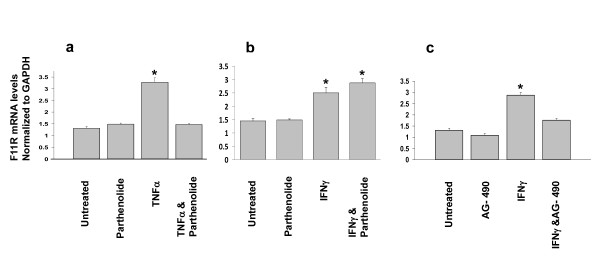
**Upregulation of F11R mRNA expression by TNFα and INFγ in endothelial cells: inhibition by the NF-kB blocker and JAK protein kinase inhibitor**. **Panels (a) and (b)**. Confluent monolayers of HUVEC were pretreated (or Untreated) for 1 hr at 37°C with the NF-kB inhibitor, parthenolide (50 μM, final concentration), added to growth supplement-free media. The proinflammatory cytokines, TNFα (100 u/mL) or IFNγ (200 u/ml), were added to the media, and the cells were incubated at 37°C for an additional 24 hrs (see TNFα & Parthenolide in Figure 4a, and IFNγ & Parthenolide in Figure 4b). The response of cells exposed only to TNFα alone (100 u/ml) is shown in the histogram displayed in Figure 4a, and the response of cells exposed only to IFNγ alone is shown in Figure 4b. The F11R mRNA levels were measured by Real-time PCR performed in triplicate for each condition. Values are the mean ± SEM. * P < 0.05 level of significance observed between ECs exposed to TNFα or IFNγ alone vs ECs not exposed to TNFα/INFγ or ECs previously treated with parthenolide followed by their exposure to cytokines. Figure 4c demonstrates the upregulation of F11R mRNA in endothelial cells by IFNγ and its inhibition by the JAK protein kinase inhibitor, AG-490. Confluent monolayers of HUVEC were either Untreated or treated with the JAK protein kinase inhibitor AG-490 (50 μM) alone (AG 490) added to growth supplement-free media and incubated for 1 hr at 37°C. The response of cells that were exposed to the cytokine IFNγ alone is depicted in the histogram IFNγ. The response of cells that were pretreated with AG 490 for 1 hr followed by their exposure to IFNγ (200 u/mL) for an additional 24 hrs is depicted in histogram labeled IFNγ & AG-490. The F11R mRNA levels were measured by Real-time PCR performed in three separate experiments, in triplicate, for each condition. Values are the mean ± SEM. * P < 0.05 significance differences in F11R mRNA in ECs exposed to IFNγ alone vs untreated ECs or ECs treated with AG-490 alone or ECs previously treated with AG-490 followed by their exposure to IFNγ

We examined whether the observed increases in the level of F11R mRNA in inflamed endothelial cells resulted from the *de novo *expression of F11R by conducting experiments involving the pretreatment of endothelial cells with the RNA synthesis inhibitor actinomycin D (5 μg/ml). Endothelial cells were pretreated (or not pretreated) with actinomycin D for a period of 1 hr at 37°C prior to their exposure to either TNFα or IFNγ. Cells that were not pretreated with actinomycin (ActD) demonstrated a significant increase in the level of F11R mRNA following their exposure to TNFα, as shown in Figure 3a (TFNα), whereas cells pretreated with ActD were unable to demonstrate the induced increase in the level of F11R mRNA induced by TNFα treatment, and a complete inhibition was observed (see TNFα & ActD). Pretreatment of cells with actinomycin D alone did not produce a decrease in basal levels of F11RmRNA (see ActD) as identical values to the basal levels measured in untreated cells were obtained. Similar to the results observed with TNFα, venous cells treated with IFNγ (200 u/ml) (as shown in Figure 3b, IFNγ) demonstrated a significant rise in their level of F11R mRNA; such an increase in F11R mRNA level could be completely blocked by the presence of ActD (see Figure 3b, IFNγ & ActD),

Next, a series of experiments utilizing specific inhibitors were examined for the potential involvement of specific pathways in the up-regulation of the F11R gene. As shown in Figure 4 (panel a), venous endothelial cells exposes to TNFα alone demonstrated a significant increase in mRNA level - however, pretreatment of these cells with parthenolide (50 μM), an inhibitor of the function of NF-κB, prior to their exposure to TNFα (see TNFα & Parthenolide), resulted in a complete blockade of their ability to up-regulate the F11R gene in response to TNFα. In the presence of the inhibitor, parthenolide, the level of F11R mRNA in cells exposed to TNFα remained unchanged (see TNFα & Parthenolide) from baseline values measured in cells not exposed to TNFα (see "untreated"), or cells treated with only the inhibitor parthenolide (see "Parthenolide"). In contrast, the blockade by parthenolide of the induction of the F11R gene by TNFα (as shown in Figure 4, panel a) was not observed in venous cells exposed to IFNγ (see Figure 4b, IFNγ & Parthenolide). Indeed, the presence of the same concentration of pathenolide did not prevent IFNγ from inducing an increase of F11R mRNA in HUVEC, and a further rise in the level of F11R mRNA could be detected in response to IFNγ in the presence of parthenolide. A possibility of cross-regulation of the IFN-γ pathway by TNFα may account for the enhanced IFN-γ responses observed in this study.

Since the inhibition of the activity of NFκB by parthenolide did not block the increase in the level of F11R mRNA induced by IFNγ, we examined whether the IFNγ-induced increase in the level of F11R mRNA could be blocked by AG490, a known inhibitor of the Jak/Stat pathway. We observed that the increase in the F11R mRNA level induced by the exposure of venous cells to the cytokine IFNγ was blocked by the pretreatment of venous cells with tyrphostin AG-490 (50 μM), the JAK protein kinase inhibitor, as shown in Figure 4 (panel c) (see IFNγ & AG-490).

### Synthesis and release/shedding of F11R by inflamed endothelial cells

**Figure 5:**
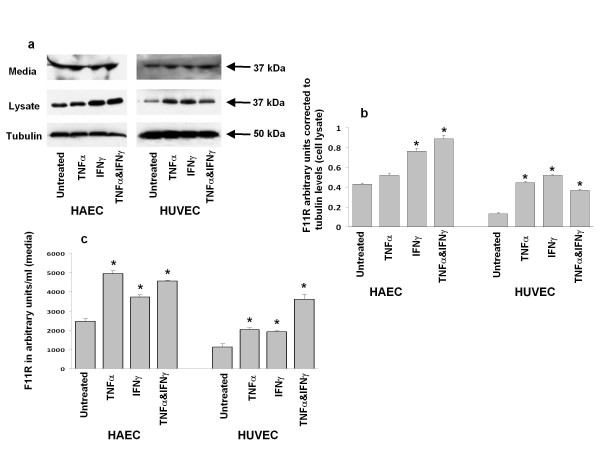
**F11R protein expression in endothelial cells treated with TNFα and INFβ**. **(a). Immunoblotting**: HAEC or HUVEC cells were treated with TNFα (100 u/mL), IFNγ (200 u/mL) or TNFα (100 u/mL) and IFNγ (200 u/mL) for 24 hrs. Collected media and cell lysates were examined for the presence of the F11R protein by SDS-PAGE (10%) followed by immunoblotting utilizing antibodies against F11R and tubulin (protein loading control, 50 kDa). (b). Quantitation of immunoblots - cell lysates. Enhanced expression of the F11R protein in cytokine-treated human aortic endothelial cells (HAEC) and umbilical vein endothelial cells (HUVEC). Quantitation of the F11R protein in cell lysates of the TNFα and/or IFNγ-treated HUVEC and HAEC, as detailed in the legend of Figure 5a. Immunoblots derived, following SDS-PAGE, were immunostained utilizing an F11R antibody. The level of the immunostained F11R protein band, of 37 kDa, was normalized to tubulin, the loading protein control, of 50 kDa. Values represent the mean ± SEM. * P < 0.05. (c). Quantitation of immunoblots - cell media. Quantitation of the F11R protein detected in the cell culture media of TNFα and/or IFNγ-treated HUVEC and HAEC (as detailed in the legend of Figure 5a), normalized to input volume. Values represent the mean ± SEM. * P < 0.05.

Previous studies have reported an enhanced presence of a soluble form of F11R (termed sF11R) in the circulation of cardiovascular patients [17] possibly due to the state of inflammation of the diseased blood vessels. As our study involved the treatment of cultured endothelial cells with inflammatory cytokines, we examined the possibility that such cytokine-treatment may result in the release/shedding and/or secretion of the F11R protein. Figure 5 shows the results of experiments designed to identify, by use of F11R specific antibody, the level of F11R in the media and lysates of inflamed endothelial cells. Figure 5a demonstrates that the F11R protein was present in the media collected from untreated venous and arterial endothelial cells. The arrow points to the immunostained F11R band calculated as a protein of molecular mass of 37 kDa. Following the treatment of these cells with TNFα and/or IFNγ, the F11R molecule continued to be detected in the media as a protein of 37 kDa. Analysis of cell lysates for the presence of F11R indicated that F11R could be detected in untreated venous and arterial cells (prepared as cell lysates) as a protein of 37 kDa, and following the treatment of venous and arterial endothelial cells with TNFα and/or IFNγ, F11R continued to be recognized as a protein of 37 kDa. Results of the quantitation of the level of the F11R protein in the cell lysates and in the media of these endothelial cells are shown in Figurs 5b and 5c, respectively. As shown in Figure 5b (for cell lysates), the level of the F11R protein found within the cell lysates of venous endothelial cells (HUVEC) was significantly elevated (> 3.5 fold) following their exposure to TNFα and/or IFNγ. In arterial endothelial cell (HAEC) lysates, a small incremental increase in F11R was observed in response to TNFα, although a significant increase (1.5X) in the F11R level was observed in response to IFNγ, with a further increase of F11R measured in cell lysates of arterial cells treated with both TNFα & IFNγ (Figure 5b).

The quantitation of the level of the F11R protein, detected as the 37 kDa protein in the cell culture media obtained from inflamed venous and arterial endothelial cells, is shown in Figure 5c. Culture media obtained from untreated HUVEC demonstrated a low, basal level of F11R. Following the treatment of HUVEC with either TNFα or IFNγ, the level of the F11R protein was significantly enhanced (2X) in the media of these cells. In the presence of both TNFα and IFNγ, a further doubling in the F11R level was observed in the media of these cells. Arterial endothelial cells (HAEC) followed a similar trend in F11R enhancement in the media in response to cytokines as that observed with media from inflamed venous endothelial cells. Approximately twice the amount of F11R was measured in the media of untreated HAEC as compared to HUVEC. The treatment of arterial endothelial cells with TNFα resulted in a significant, 2.5-fold increase in the level of F11R detected in the media, with approximately a 1.5-fold increase in F11R detected in the media of IFNγ-treated cells. The simultaneous treatment bothTNFγ & IFNγ resulted in a 2-fold increase in F11R protein in the media of these cells, levels similar to those observed with either TNFα or IFNγ alone.

### Effects of the silencing of the F11R gene: blockade of F11R protein expression in endothelial cells

To determine directly whether the F11R protein is a critical molecule involved in the adhesion of platelets to endothelial cells, the expression of the F11R gene was silenced in inflamed endothelial cells by utilizing small interfering RNAs, F11R siRNAs. Transfected endothelial cells then were examined for their ability to recruit freshly-isolated human platelets in platelet-adhesion experiments. However, prior to this series of experiments, we determined the degree of knockdown of the F11R gene due to the transfection of venous and arterial endothelial cells by F11R siRNA: indeed, we observed that 82% knockdown of F11R occurred in HUVEC, and a 72% knockdown of F11R occurred in HAEC.

**Figure 6:**
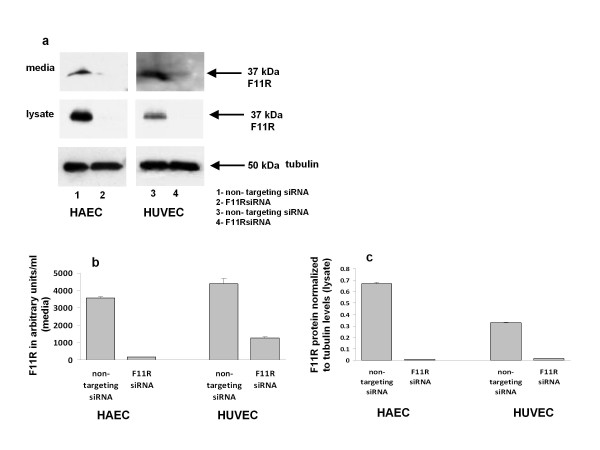
**Expression of the F11R protein in inflamed endothelial cells: silencing of the F11R gene in HAEC and HUVEC using F11R siRNA**. (a). Immunoblots demonstrate the detection of the F11R protein retained in cells (cell lysates) and released into the media of inflamed HAEC and HUVEC. Both aortic and umbilical vein endothelial cells were transfected with either the control, non-targeting siRNA or by the specific F11R targeting siRNA (as detailed in the Material and Methods section). Subsequently, the cells were treated with the proinflammatory cytokines TNFα (100 u/ml) and IFNγ (200 u/ml) for 24 hrs, followed by SDS-PAGE and immunoblotting utilizing F11R antibody (arrows point to F11R), and tubulin, as the protein loading control, of 50 kDa. Lanes 1 and 3 depict the F11R protein as detected in cytokine-treated HAEC or HUVEC transfected with the nontargeting siRNA. Lanes 2 and 4 depict the F11R protein as detected in cytokine-treated HAEC and HUVEC transfected with the specific targeting F11R siRNA.(b). Quantitation of immunoblots of the immunostained F11R protein, detected in the cell culture media of HAEC and HUVEC endothelial cells transfected with either the non-targeting siRNA or the specific targeting F11R siRNA, followed by the exposure of transfected HAEC and HUVEC to a combination of the proinflammatory cytokines TNFα (100 u/ml) and IFNγ (200 u/ml) for 24 hrs. The values for F11R were normalized to tubulin levels by dividing the integrated density of the specific band by the integrative density of the tubulin band. ANOVA statistical analysis was performed on the normalized values. All values are the average of three immunoblots ± SEM. (c). Quantitation of the immunostained F11R protein within the cell lysates of HAEC and HUVEC transfected with either the non-targeting siRNA or the specific targeting F11R siRNA, and further treated with the proinflammatory cytokines TNFα (100 u/ml) and IFNγ (200 u/ml) for 24 hrs. F11R-immunostained protein bands were quantified by normalization to tubulin using image J. The F11R values were normalized to tubulin. ANOVA was performed on the normalized value (n = 3). Values depict the mean ± SEM, * p < 0.005.

A comparison of the effects of transfection of endothelial cells on F11R levels in arterial (HAEC) and venous (HUVEC) endothelial cells transfected either by a nonspecific siRNA or a specific F11R siRNA is shown in Figure 6a. As shown in lane 1, the utilization of a nonspecific siRNA in the transfection of TNFα and IFNγ-inflamed arterial endothelial cells(HAEC) did not block the enhancement of the synthesis of the F11R protein which was identified both in the lysate of these arterial cells as well as in their media (see Figure 6a, HAEC, lane 1). In contrast, as shown in Lane 2, the transfection of arterial endothelial cells (HAEC) by the specific-F11R targeting siRNA resulted in the inhibition of F11R synthesis - the F11R protein was neither expressed in lysates nor detected in the media of TNFα and IFNγ-treated arterial endothelial cells (HAEC, see lane 2). Similar to the results obtained with inflamed arterial cells transfected with a non-targeting siRNA, the synthesis of the F11R protein was not blocked following the transfection of inflamed venous endothelial cells (HUVEC) by the non-targeting siRNA (see Figure 6a, HUVEC, lane 3). However, as shown in Lane 4, the F11R protein was neither expressed in the lysate nor detected in the media of TNFα and IFNγ-inflamed venous endothelial cells following the transfection of HUVEC by the specific-F11R targeting siRNA (HUVEC, lane 4). Quantitation of the F11R protein (immunostained 37 kDa) revealed that the transfection of inflamed arterial (HAEC) and inflamed venous (HUVEC) endothelial cells by specific interfering F11R siRNA resulted in a significant inhibition in the synthesis and release/shedding of the F11R protein. As shown in Figure 6b, almost 100% decrease of F11R occurred in media of F11R siRNA-transfected HAEC; an 80% decrease of F11R in the media of F11R siRNA-transfected HUVEC was observed. Furthermore, the targeted transfection of TNFα and IFNγ-treated HAEC and HUVEC by F11R siRNA resulted in the complete inhibition of F11R expression in the cell lysates of these inflamed arterial and venous endothelial cells as (shown in Figure 6c).

### Effects of the silencing of the F11R gene: inhibition of platelet adhesion to inflamed endothelial cells

**Figure 7:**
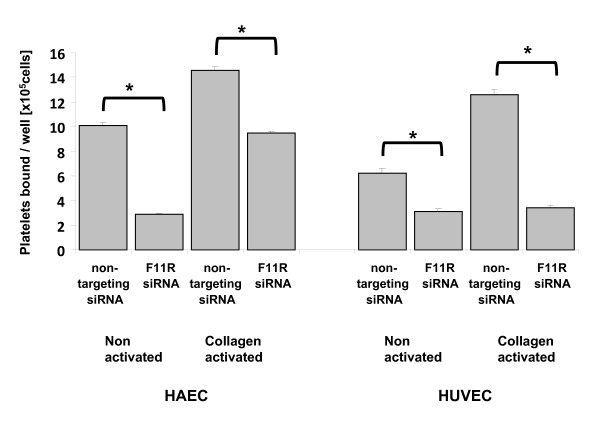
**Blockade of platelet adhesion to inflamed human aortic (HAEC) and human umbilical endothelial vein endothelial cells (HUVEC) by F11R siRNA: inhibition by silencing of the F11R gene**. Transfection of HUVEC and HAEC was conducted by using either the non-targeting siRNA or the F11R targeting F11R siRNA, as detailed in the Material and Methods section. Following transfection, both HAEC and HUVEC were pretreated with a combination of cytokines TNFα (100 u/ml) and IFNγ (200 u/ml) for 24 hrs. Afterwards, suspensions of either non-activated or collagen-activated platelets (as detailed in the Material and Methods section) were applied unto monolayers of the inflamed ECs, and the adhesion of platelets to the cytokine-treated ECs was monitored. The values represent the adjusted means ± SEM for the number of platelets bound to the ECs/per well from 5 separate experiments. * P < 0.05.

To examine the functional consequences resulting from the silencing of the F11R gene and inhibition of F11R protein expression by specific targeting of the F11R gene in endothelial cells, we examined whether the transfection by F11R siRNA altered the ability of cytokine-inflamed endothelial cells to attract and bind human platelets. In this investigation, both the adhesion of nonactivated platelets as well as platelets activated by collagen, a potent platelet agonist, were examined. As shown in Figure 7 for HUVEC, the transfection of venous endothelial cells by F11R siRNA resulted in a significant reduction (by 50%) in the adhesion of non-activated platelets to F11R siRNA- transfected HUVEC exposed to cytokines TNFα and IFNγ, although the ability of platelets to bind to inflamed HUVEC transfected with the non-targeting siRNA remained intact. Furthermore, the transfections of HUVEC by F11R siRNA significantly inhibited the ability of collagen-activated platelets to bind to the inflamed HUVEC, although HUVEC transfected with the nontargeting siRNA demonstrated a high degree of binding of platelets. Similarly, both non-activated as well as collagen-activated platelets exhibited a high degree of adhesion to arterial endothelial cells (HAEC) transfected with the non-targeting siRNA (Figure 7). However, the silencing of the F11R gene of HAEC by transfection with F11R siRNA produced significant effects on the ability of platelets to adhere to these cells. As shown in Figure 7, a significant blockade of the adhesion of non-activated platelets as well as collagen-activated platelets was observed following the transfection of the inflamed HAEC by F11R siRNA.

## Discussion

The results reported here provide direct evidence for the critical role of F11R in the initiation of atherogenesis. This study demonstrates that inhibition by specific siRNA of the *de-novo *biosynthesis of F11R, induced in endothelial cells by inflammatory cytokines, significantly inhibits the adhesion of human platelets to inflamed endothelial cells, an adhesion that would lead to production of atherosclerotic plaques in non-denuded blood vessels [3].

Under physiological conditions, the non-activated, healthy endothelium expresses low levels of F11R- mRNA and the F11R/JAM-A protein resides primarily within the endothelial tight junctions [6]. Under these conditions, circulating human platelets that constitutively express the F11R protein on their cell surface ^4 ^do NOT adhere to a non-inflamed endothelium [3]. On the other hand, when endothelial cells are exposed to the proinflammatory cytokines TNFα and/or IFNγ, F11R- mRNA levels rise significantly, followed by increased *de-novo *synthesis of the F11R-protein and the insertion of newly-synthesized F11R molecules into the luminal surface of the endothelium [18]. The present study provides direct evidence for the progression of this chain of events by the use of two blockers of mRNA synthesis: Actinomycin, an overall inhibitor of RNA synthesis, and F11R-siRNA, a specific inhibitor of the synthesis of F11R-mRNA. Both of these inhibitors blocked the enhancement of expression of F11R-mRNA and of the synthesis of the F11R protein in cytokine-stimulated arterial and venous endothelial cells. Most importantly, the critical pathophysiological role of the F11R-protein in the formation of a thrombogenic surface was proven by demonstrating that the inhibition of the expression of F11R-mRNA and thus of the increase in F11R protein in cytokine-exposed endothelial cells prevents the adherence of human platelets to inflamed endothelial cells.

Ozaki et al. [19], were the first to report the changes in the localization of JAM/F11R protein in human umbilical vein endothelial cells that were treated simultaneously with the cytokines TNFα and IFNγ. As this treatment caused a disappearance of JAM from intercellular junctions, but no change in the total level of the protein [19], the authors concluded that the exposure of endothelial cells to cytokines causes a redistribution of this protein from intercellular junctions to the surface of the plasma membrane of the inflamed endothelium. Our present results demonstrate that such treatment of arterial and venous endothelial cells with the cytokines TNFα and IFNγ induces *de-novo *biosynthesis of F11R-mRNA and of the F11R protein. Taken together, all the data indicate that the lack of change in overall levels observed in the redistribution of the F11R/JAM protein in inflamed EC involve the disappearance of F11R/JAM-A molecules of the intercellular junctions that are degraded and/or released to the circulation (as discussed below). These are replaced with newly synthesized molecules of F11R/JAM-A that are inserted into the luminal side of the plasma membrane, that then acquires a thrombogenic surface.

As reported here, the biochemical pathway leading to the upregulation of the F11R gene following exposure of endothelial cells to the cytokine TNFα involves the NF-κB signaling pathway. Parthenolide, an inhibitor of NF-κB, blocked the TNFα-induced expression of the F11R gene - results consistent with our findings of NF-κB binding-sites in the promoter region of the F11R gene [11]. On the other hand, the upregulation of F11R mRNA by IFNγ was blocked solely by the antagonist AG-490, a JAK tyrosine kinase inhibitor, indicating the involvement of the JAK/STAT signaling pathway in the induction of F11R mRNA and the *de-novo *expression of the F11R protein by IFNγ. As the analysis of F11R gene structure indicates the presence of two promoters with regulatory elements consisting of NF-κB, GATA, Inr, ets sequences, TATA, and several GC and CCAAT boxes [11], thus it is the participation of these regulatory elements that may account for the effects of IFNγ on the induction of F11R mRNA and protein observed here.

An additional important result of the present report is that exposure of endothelial cells to the inflammatory cytokines TNFα and IFNγ results in the release of soluble F11R molecules (sF11R) into the extracellular medium. Thus, the release of F11R appears to be an integral part of the pathological process induced within the vasculature in response to inflammatory cytokines. The important clinical implications of this process were reported previously [17,20]. A significant increase in the level of sF11R was found in the serum of patients with coronary artery disease (CAD) associated with high risk of atherosclerosis and heart attack [17]. Furthermore, in this study the levels of serum-sF11R correlated significantly with the clinical severity of the disease [17]. In other clinical studies, Salifu et al. [20] reported of significantly enhanced levels of sF11R in the plasma of renal disease patients prone to atherosclerosis, and Ong et al. [21] have demonstrated enhanced levels of sF11R in the serum of hypertensive patients. An increase in the level of the cytokine TNFα was also determined in the circulation of CAD patients and hemodialysis patients [17] and these levels correlated positively with the circulating levels of sF11R. We have proposed that increased levels of sF11R immunoreactivity in plasma or serum can serve as markers for the initiation and progression of atherosclerosis. Similar to the results observed with HAEC and HUVEC, recent studies [22] have shown that the exposure of cultured primary or immortalized human brain microvascular ECs to proinflammatory cytokines resulted in a decrease of F11R immunostaining at the tight junctions. However, the serum levels of sF11R were NOT altered in patients with multiple sclerosis and ischemic stroke that have demonstrated an inflamed blood-brain barrier. Haarmann et al. [22], suggest that ECs of the blood-brain barrier are not induced to release sF11R by inflammatory stimuli, and that this resistance serves as a unique protection of the CNS compartment.

Potential mechanisms by which inflammation may lead to the formation of F11R detected in the plasma or serum of cardiovascular patients may involve the shedding of endothelial cell membrane-microparticles, as-well-as the release of soluble fragments of F11R by the action of circulating extracellular proteases. The occurrence of both these types of events have been previously reported. In early studies reported in 1986, we have demonstrated that exposure of human platelets to granulocytic elastase (released during inflammation) results in the release of soluble fragments of the platelet fibrinogen receptor, α_2_β_3 _integrin, and consequently in the direct binding of fibrinogen and the aggregation of platelets by fibrinogen [23]. Evidence for the potential involvement of the disintegrin- metalloproteases in the proteolytic cleavage of JAM-A was provided by Koenen et al. [24], who detected a soluble form of the F11R/JAM molecule with molecular mass of 33kDa in the conditioned media of inflamed HUVEC *in culture*, as well as *in-vivo *in cytokine-treated mice [24]. The generation of endothelial-membrane microparticles has been reported by Combes et al. [25] and by VanWijka et al. [26]. Thus, the shedding of F11R-containing microparticles from platelets and endothelial cell membranes, and the action of proteases degrading the protein in intercellular junctions of EC that disappear during inflammatory processes, and/or on the surface of the plasma membrane of platelets, may all represent alternate mechanisms operating during inflammatory processes that are responsible for the appearance of soluble and microparticle-bound F11R molecules in the plasma and serum of patients with cardiovascular diseases.

We previously have shown that significant levels of the F11R mRNA and protein are expressed in vessels of CAD patients exhibiting clinical symptoms of coronary artery disease associated with atherosclerotic plaques [18]. The increased expression of F11R at sites of atherosclerotic lesions was shown by others to be highest in unstable atherosclerotic plaques [27], thereby demonstrating the involvement of F11R in both atherogenesis and atherothrombosis.

We have previously identified three different types of cells present in the atherosclerotic plaque express high levels of F11R. These are platelets, endothelial cells and smooth muscle cells [4,28]. Accordingly, the pathophysiological functioning of the F11R protein was examined for each cell type, and demonstrated to involve platelet-endothelial cell adhesive interactions, platelet aggregation, and the migration and proliferation of cytokine-stimulated smooth muscle cells. Stellos et al. [29] reported a role for the F11R in the repair of the injured, inflamed endothelium, by showing that JAM-A/F11R molecules expressed on endothelial progenitor cells are required for the re-endothelialization of the vasculature, yet another critical role for F11R. Our previous studies utilized two F11R peptide-antagonists to determine that F11R provides well over 50% of the adhesive force operating between platelets and inflamed EC [9]. The involvement of JAM-A in neointima formation following wire-injury of carotid arteries was reported by Zernecke et al. [30]. Interactions between activated platelets, through their release of the chemokine RANTES, and its deposition onto endothelial cells were shown to be dependent on JAM-A [30]. The results of the present study obtained with an experimental approach that specifically silences the F11R gene, provide direct evidence for the critical role of F11R in the adhesion of platelets to the endothelium under inflammatory conditions, which is an early, initial stage of plaque formation in atherogenesis. Accordingly, we propose that specific antagonists of the pathological actions of F11R represent a new target for the development of novel drugs for the prevention and treatment of atherosclerosis, heart attacks, stroke, and other cardiovascular disorders triggered by inflammatory processes.

## Conclusion

We conclude that the transcription and translation of the human F11R gene are required initial steps of atherogenesis induced by inflammatory cytokines in the vasculature, leading to atherosclerosis, heart attacks and stroke.

## Competing interests

The authors declare that they have no competing interests.

## Abbreviations

(BCA): Bicinchoninic acid; (DMSO): dimethyl sulfoxide; (ECs): endothelial cells; (EDTA) acid: ethylenediaminetetraacetic; (EGTA): ethylene glycol tetraacetic acid; (F11R): F11 receptor; (FCS): fetal calf serum; (GAPDH): glyceraldehyde-3-phosphate dehydrogenase; (HAEC): Human Aortic Endothelial Cells; Human Umbilical Vein Endothelial Cells (HUVEC); (IFNγ): interferon gamma; (JAK/STAT): Janus kinase/signal transducer and activator of transcription; (JAM-A): junctional adhesion molecule-A; (LSGS): low serum growth supplement; (mRNA): messenger ribonucleic acid; (NF-κB): nuclear factor kappa-B; (PBS): phosphate buffered saline; platelet rich plasma (PRP); (SDS): sodium dodecyl sulfate; (SDS-PAGE): sodium dodecyl sulfate-polyacrylamide gel electrophoresis; (siRNA): short interfering RNA; (TNFα): tumor necrosis factor-alpha; (AG-490): tyrphostin, tyrosine kinase inhibitor.

## Authors' contributions

BMA: Participated in design of studies, carried out all experiments and was involved in the drafting of the manuscript. These studies constitute a partial requirement for the attainment of her PhD in the Department of Medicine and Cell Biology/Anatomy.

JDM: Has made significant contributions to the conception and interpretation of the data.

MOS: Has made significant contributions to this work, has participated in analysis and interpretation of data, has performed the statistical analysis and was involved in drafting of the manuscript.

EK: Has been involved in experimental design, data analysis, the writing of the manuscript. Was critically important for the intellectual content of this work, and has given final approval of the version to be published.

YHE: Has been involved in experimental design, data analysis, the writing of the manuscript, and critically important for intellectual content of this work.

AB: Has made significant contributions to the conception, design and supervision of all experiments, performed data analysis and interpretation of data, supervised and coordinated all studies, and drafted the manuscript. All of the authors have read and approved the manuscript.
